# Development and evaluation of a dual-purpose machine for chopping and crushing forage crops

**DOI:** 10.1016/j.heliyon.2023.e15460

**Published:** 2023-04-15

**Authors:** Hossam El Ghobashy, Yousry Shaban, Mahmoud Okasha, Solaf Abd El-Reheem, Mohamed Abdelgawad, Rania Ibrahim, Heba Ibrahim, Khaled Abdelmohsen, Mahmoud Awad, Mokhtar Cottb, Mohamed Elmeadawy, Wael Fathy, El-Sayed Khater

**Affiliations:** a Agricultural Engineering Research Institute (AEnRI), Agricultural Research Center (ARC), Giza 12611, Egypt; b Agricultural and Biosystems Engineering Department, Faculty of Agriculture, Benha University, Kalubia 13736, Egypt

**Keywords:** Forage machine, Maize chopper, Efficiency, Productivity, Specific energy

## Abstract

Reducing reliance on fossil fuels with clean and sustainable alternatives is essential for mitigating climate change and global warming-related environmental concerns. Previous researchers have studied the performance of choppers and crushers as separate units powered by diesel or gasoline engines. Nowadays, an increasing interest in producing Eco-friendly machines that stand out for being dual purposes, cost-effectiveness, and with lengths suitable for feeding ruminants are imperative to achieving economic and sustainable goals. Therefore, this study aims to solve these issues and gaps by developing and evaluating a dual-purpose forage machine for chopping and crushing operations to achieve both operations more efficiently and at a lower cost. The developed forage machine's performance was evaluated for chopping operation using maize stalks with four different rotational speeds of 1200, 1400, 1600, and 1800 rpm and four different moisture contents of 22.7, 43.3, 59.8, and 74.6% (w.b.). Also, the crushing operation was evaluated using maize ears with four different crusher speeds of 1200, 1400, 1600, and 1800 rpm and three different sieves with holes' diameters of 6, 8, and 10 mm. The results concluded that the highest efficiencies with values of 94.17 and 92.85% were obtained at 1800 rpm chopper rotational speed and 22.7% moisture content for the chopper and 1200 rpm crusher rotational speed and 10 mm sieve hole diameter for the crusher, respectively. At these proper operational parameters, the machine productivity of 2.44 and 0.31 ton.hr^−1^, the specific energy requirements of 3.22 and 4.50 kW h.ton^−1^, and the estimated production costs of 23.56 and 121.24 EGP.ton^−1^ (1.25 and 6.38 USD.ton^−1^) were obtained for chopper and crusher, respectively.

## 1 Introductions

Typically, small-scale and marginal farmers will breed some poultry, goats, buffalo, and/or dairy farming as an additional source of income to support their income in case of crop failure. In addition, the world energy crisis will encourage using livestock as bioenergy and recycling waste into organic manure [1]. Under the current conditions, the world is striving to manage agricultural waste and add economic and environmental value to it. In this trend, Teixeira et al. [2] reported that lean manufacturing demonstrated efficacy as a management concept for eliminating waste from production processes, leading to operational gains and economic returns. Crop residues and agro-industrial by-products are two major feed resources for all ruminants, and they play an important role in ruminant nutrition [3]. An adult bullock consumes 10–15 kg of fodder daily, while cows and buffalo consume 8–10 kg daily 1. Agricultural residues are one of the most plentiful forage resources in Egypt and the world. The total residue produced in Egypt was estimated to be approximately 30 million tons annually. The largest share from the previous percentage is represented by wheat straw, with a percentage of 37.7%, followed by maize stalks, with a percentage of 16.6%, and rice straw, with a percentage of 10.7% [4]. Chopping is required for either use, and reducing the shearing force has been regarded as one of the most efficient methods to save energy [5]. Both chemical and physical methods are used to improve the quality of crop residues and roughages. The physical treatment of residues, which comes before the chemical treatment, enhances the material's acceptance response to the chemical treatments. Chopping, shredding, grinding, and pelleting are examples of physical treatment. Grinding and pelleting fibrous materials increase the surface area exposed to microbial attack and accelerate the flow rate of digests via the gastrointestinal tract [6]. Chaffing fodders and straws into small pieces before feeding to animals enhances digestibility, palatability and preserves energy used in mastication. Cutting fodder techniques can be done manually, using a diesel-powered machine, or with an electric-powered machine [7].

A new power-operated cylindrical type chaff cutter cum grinder (1.5 kW) was developed to avoid the problems associated with manual fodder cutting. It consists of a power transmission unit, a main frame, a feeding hopper, a cutting unit, a head unit, and a discharge unit. The machine productivities for wet fodder were 330 and 272 kg h^−1^, but for dry fodder were 202.9 and 174.9 kg h^−1^ for sorghum and maize, respectively. In addition, the grinder productivity was 100 and 95 kg h^−1^ for the sorghum and maize grains, respectively. The machine's chaffing efficiencies were 86.40 and 84.15% for wet fodder but were 86.30 and 80.50% for dry fodder for sorghum and maize, respectively. The grinder efficiencies were 95 and 96% for the sorghum and maize grain, respectively [1]. The chopper cum grinder is a dual-purpose machine for cutting straws and grinding the animal feed grains such as corn, wheat, and others. A chaff cutter is a mechanical system that chops straw or hay into small, equal pieces that may be blended with other forage grass to feed farm animals. This aids digestion and keeps animals from rejecting any component of their food. A grinder is a machine that mills grain into coarse flour, using repeated blows from many hammering plates to feed livestock [8].

A local thresher machine was modified to mince and chop agricultural leftovers, such as rice straw and corn, with the least energy possible. The modified machine operated with two types of blades (free sharp blades + serrated discs). The modified machine performance was assessed in terms of productivity and cutting efficiency for two different crops of corn stalk and rice straw under three operating speeds of 1200, 1600, and 2000 rpm at three moisture contents of 8, 10, and 12%. The results showed that the highest machine productivity and the optimal cutting efficiency were 720 kg h^−1^ and 95.8% for corn stalks. In contrast, in the case of rice straw, the results recorded 490 kg h^−1^ and 91.6% at a rotational operating speed of 2000 rpm and 8% moisture content. Cutting force can be increased during the operation using disc mills and other flail blades [9]. In the same trend, Hegazy et al. [10] modified a star forage chopper machine (SFCM) to reduce the power required for forage chopping and to improve forage cutting efficiency. The SFCM's performance was assessed based on cutting rice straw, and cotton stalks at varying feed rates and knife speeds. The minimum cut length for rice straw was 13.5 and 12.7 mm for cotton stalks, and they were achieved by using a 1500 kg h^−1^ feed rate with 136.2 m s^−1^ knives speed with toothed blades and toothed blades normal (flail) blades, respectively. The minimum power required to cut rice straw and cotton stalks were 1.81 and 1.76 kW and were achieved by using 800 kg h^−1^ feed rate with 78.6 m s^−1^ knives speed for toothed blades and normal (flail) blades, respectively. Along the same lines, Awad et al. [11] manufactured and evaluated the performance of a combined machine for collecting and chopping rice straw to preferred lengths in feeding ruminants.

The small crushing machine performance was evaluated for faba bean at a moisture content of 9.5% (d.b.) using various hammer drum speeds of 1000, 1250, 1500, and 1750 rpm, feeding rates of 60, 90, and 120 kg h^−1^, and sieves holes diameters of 2, 4 and 6 mm. The results revealed that the maximum productivity of 118 kg h^−1^, maximum machine efficiency of 99%, and lowest specific energy of 7.50 kW h.ton ^−1^ were achieved at 1750 rpm, 6 mm, and 120 kg h^−1^ for hammer drum speed, sieve hole diameter, and feeding rate, respectively. While the optimum result for crushed faba bean particle size was achieved at 1750 rpm, 2 mm sieve hole diameter, and a feeding rate of 60 kg h^−1^ [12].

Most farmers yet depend on simple hand tools (manual), particularly the sickle, for chopping forage; however, using such tools is drudgery and time-consuming, which in turn causes health risks [13]. Furthermore, Egypt's imports of agricultural machinery account for 15% of its total agricultural imports, which are estimated at around 1100 USD million [14].

The previous review concluded that manual chopping and crushing operations are arduous, time-consuming, and low-efficiency. Moreover, imported machines are very expensive, especially with the difference in currency exchange rates. Therefore, this study aims to develop and assess the performance of a locally made dual-purpose machine for forage crop chopping and crushing operations in one machine suitable for small and medium scales farms to perform both operations more efficiently at low cost.

## 2 Materials and methods

### 2.1 The chopping and crushing machine

**Fig. 1:**
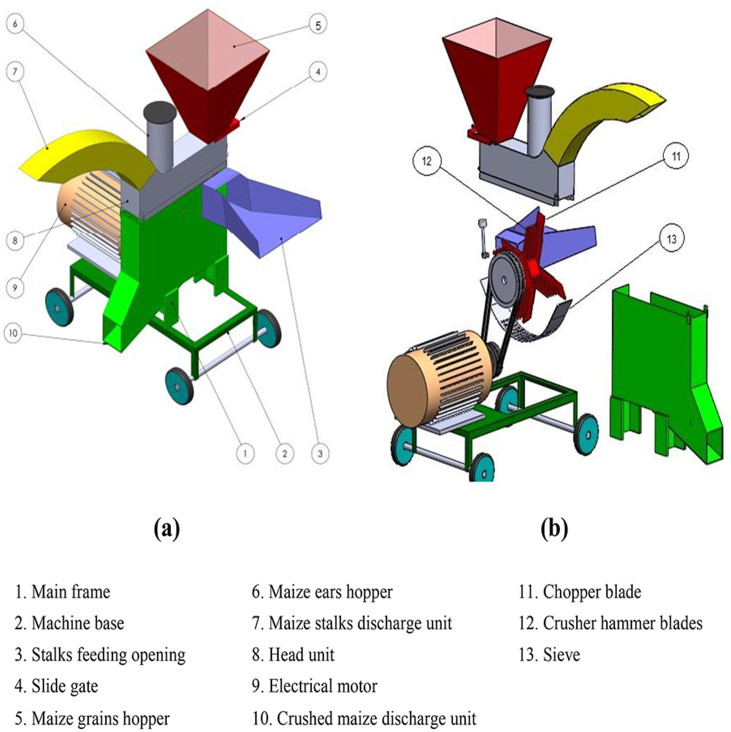
Isometric view of the chopper and crusher **(a)**, chopping and crushing mechanism with power transmission **(b)**.

**Fig. 2:**
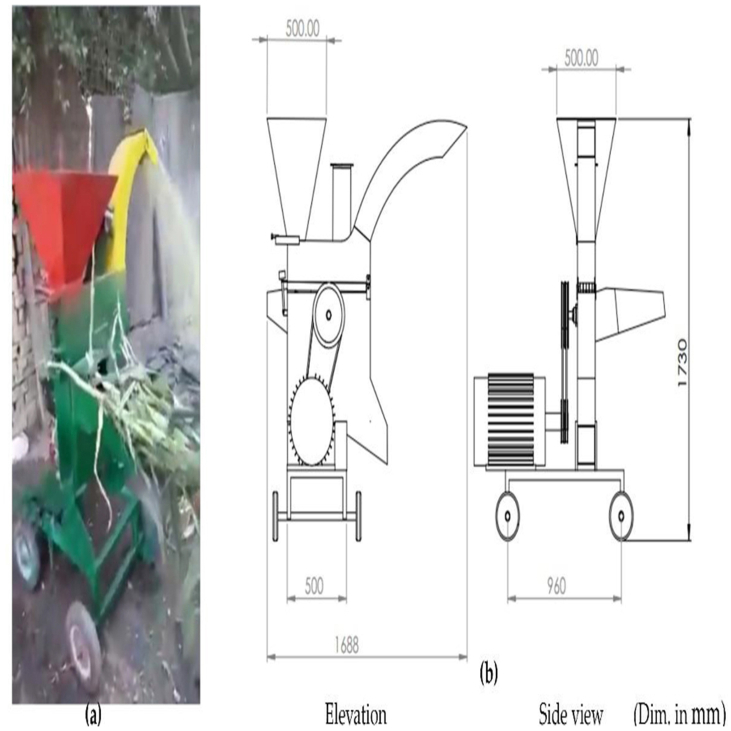
General view of forage machine during operation **(a)**, schematic diagram of the forage machine **(b)**.

A forage crop chopping and crushing machine was manufactured and developed at a private workshop in Al-Qanatir Al-Khayriyah City, Qalyubia Governorate, Egypt. In addition, the experiments of the modified machine were conducted at the Agricultural Engineering Research Institute (AEnRI), Giza, Egypt, during the summer of 2022 for the maize variety (Yellow maize single cross 176). The main components of the chopping and crushing machine are main frame, head unit, feeding hoppers, chopping and crushing units, discharge units, and power transmission, as shown in Fig. 1 (a, b). Fig. 2 (a, b) illustrates a general view and a schematic diagram of the developed forage machine.

#### 2.1.1 The machine's frame

The machine's main frame was constructed from iron bars in an L-shape (3 mm thickness and a cross-section of 40 × 40 mm), as shown in Fig. 1. The machine's frame dimensions are 1200 × 1000 × 150 mm in height, length, and width, respectively. All parts of the forage machine were constructed from anti-rust materials.

#### 2.1.2 Head unit

The head unit of the forage machine is made of two plates attached to the main frame with nut bolts. Three hoppers are made of 1.5 mm thick mild steel sheets on the head unit. The chopper hopper opening is bolted to the side plates by nut bolts, and it has sufficient inclination and dimensions of 300 × 250 × 100 mm for length, width, and height, respectively. The maize stalks are directly fed into the chopping unit. At the same time, the other two crushing hoppers for maize grains and ears are mounted above the head unit. The maize grains hopper is a trapezoidal shape, with a cross-sectional of 500 × 500 mm at the top and 250 × 250 mm at the bottom with a thickness of 2 mm, as shown in Fig. 1, Fig. 2. This inclination allows grains to flow down the hopper walls via a slide gate below the grains hopper to regulate the grains feeding from the hopper to the crushing unit. The maize ears hopper is a cylindrical shape with dimensions of 150 mm in diameter and 300 mm in length and is designed to feed the maize ears one at a time.

#### 2.1.3 Chopping and crushing units

The chopping unit comprises four fixed sharp blades evenly distributed on the axis of the rotating disc shaft, and the blades have dimensions of 250 × 80 × 5 mm for length, width, and thickness, respectively. Contact of maize stalks with rotating chopper blades causes an impact and shearing action, which cuts maize stalks into the desired length pieces. The cut pieces are routed through the discharge unit, which is located above the machine.

The crushing unit consists of four groups of fixed hammer blades; in each group, five sharp hammer blades are installed beside the chopping blades. The hammer blades have dimensions of 250 × 60 × 5 mm. The crushing unit has one sieve with holes of 6, 8, and 10 mm in diameter that can be changed to obtain the desired sizes. The particles of crushed maize ears are passed through previous sieves' diameters to the discharge unit at the machine's bottom. All movable parts or components are equipped with a protective device; no parts or components can be opened without using tools for safety purposes. The manufacturing, operation and testing processes were done based on standard procedures and with recommended guidelines and frameworks [15].

#### 2.1.4 Power source

A three-phase electric motor (7.5 kW ≈ 10.0 hp) with a 10 cm diameter pulley and motor speed of 3000 rpm is the power source of the forage machine. The power is transmitted to the chopping and crushing units using different changeable V-shaped belts and four pulleys with diameters of 25, 21.4, 18.8, and 16.7 cm to control and obtain the required speeds of 1200, 1400, 1600, and 1800 rpm, respectively.

Selecting the motor's power in this study according to Okasha [9], using Eq. (1), as follows:

(1) $$P=\frac{Tr\times N}{9550}$$

where P is power (kW); Tr is torque (N.m); N is rotational speed (rpm).

The required force for cutting the maize stalk using a universal testing machine was 0.2 N mm^−1^ per unit width [1]. Based on the above equation, a 7.5 kW electric motor is sufficient to be used in this study.

### 2.2 Machine performance evaluation

#### 2.2.1 Treatments

Chopper was evaluated using maize stalks at different rotational speeds of 1200, 1400, 1600, and 1800 rpm under different moisture contents of 22.7, 43.3, 59.8, and 74.6% (w.b.). Crusher was tested using maize ears at different sieve holes diameters of 6, 8, and 10 mm and different rotational speeds of 1200, 1400, 1600, and 1800 rpm. Furthermore, each treatment was replicated three times in this study.

#### 2.2.2 Measurements

Some characteristics in terms of plant height, stalk diameter, ear length, ear diameter, whole plant mass, plant mass without ears, one ear mass, and ear numbers per plant were determined based on 50 samples selected randomly, and their average values were 2480, 36, 265, 54 mm, 2135, 1130, 335 g, and 3 ears per plant, respectively.

Maize stalks were chopped after harvesting and at different times with different moisture contents, and maize stalk moisture contents were determined on a wet basis (w.b.) according to AOAC [16] by drying the stalk samples in a hot electrical oven at 105 °C temperature for 24 h. Machine productivity is defined as the ratio of output mass (ton) from chopping or crushing processes to machine operation time (hr), according to Mady et al. [17].

The chopping efficiency was calculated according to Habib et al. [18]. They mentioned that the appropriate stalk cutting length is between 0 <Lc < 50 mm, which can be used for composting and forage production. A 1 kg random sample of the chopped maize stalks without ears after passing through the discharge unit was taken to determine the mass of cutting length, which is less than 50 mm in the sample, using a digital caliper and attribute it to the initial mass of the sample, as follows in Eq. (2).

(2) $$\eta C=\frac{C_{m}}{C_{i}}\;x\;100$$

where ηC is the chopping efficiency (%); C_m_ is the mass of the measured lengths of the sample, which is < 50 mm (kg); C_i_ is the initial sample mass of 1 kg.

The crushing efficiency was calculated as the mean particle diameter (mm) ratio for crushed maize ears to machine sieve hole diameter. The mean particle diameter for the sample can be computed in a laboratory by sifting the sample with standard laboratory sieve sets with variable diameters and measuring the residual mass above each sieve, as follows in Eq. (3) according to Finch [19].

(3) $$D=\frac{\left(C_{1}\times M_{1}+C_{2}\times M_{2}+--+C_{n}\times M_{n}\right)}{\left(M_{1}+M_{2}+--+M_{n}\right)}$$

where D is the mean particle diameter (mm); C is the diameter of the sieve (mm); M is the residual mass above the sieve (g); n is the sieve number.

The required electric power (P) in this study was calculated according to Ibrahim [20], as follows in Eq. (4).

(4) $$P=\sqrt{3}\times I\times V\times \eta_{m}\times \mathrm{Cos}\;\theta /1000$$

where P is the power requirement for chopping and crushing operations (kW); I is the line current strength (Amperes); V is the potential difference (Voltage) being equal to 390V; η_m_ is the mechanical efficiency ≈ 95%; Cos θ is the power factor ≈ 0.84.

The specific energy (SE) for chopping and crushing operations was estimated using Eq. (5).

(5) $$SE=\frac{P}{MP}$$

where SE is the specific energy (kW.hr.ton^−1^); P is the required power (kW); MP is machine productivity (ton.hr^−1^).

The machine's total cost includes two categories: fixed cost (depreciation, interest, shelter, taxes, and insurance) and variable cost (repair and maintenance, energy cost, labor cost), according to Suliman [21], as follows in Eqs. (6), (7), (8), (9), (10), (11), (12), (13), (14), (15).

(6) $$D=\frac{P-Sr}{Le}$$

where D is the depreciation cost (EGP per year); P is the machine price (≈17000 EGP); Sr is salvage rate (10% from the machine price, EGP); Le is the machine life expectancy (≈5 years).

(7) $$I=\frac{P+Sr}{2}\times i$$

where I is the interest cost (EGP per year); P is the machine price (EGP); Sr is salvage rate (EGP); i is the interest as compounded annually (≈10%)

The machine's shelter, taxes, and insurance were assumed to be 4.5% of the machine's purchase price. Yearly operating hours are 300 h and 500 h for the chopper and crusher, respectively. The exchange rate is 1 USD ≈19 EGP

(8) $$Fixed\;cost\;\left(EGP\;h^{-1}\right)=\frac{D+I+0.045P}{Yearly\;operating\;hours}$$

(9) $$RM=\frac{100\%D}{Yearly\;operating\;hours}$$

where RM is repair and maintenance cost (EGP per year); D is the depreciation cost (EGP);

(10) E=Ec×Ep

where E is the energy cost (EGP.hr^−1^); Ec is the total consumed electric power (kW); Ep is the energy price (≈1.20 EGP.kW.hr^−1^);

(11) Lc=Ow×N

where Lc is the labor cost (EGP.hr^−1^); Ow is the operator's hourly wage (≈10 EGP.hr^−1^); N is number of operators (one operator);

(12) $$Variable\;cost\;\left(EGP.{hr}^{-1}\right)=RM+E+Lc$$

(13) $$Total\;cost\;\left(EGP.{hr}^{-1}\right)=Fixed\;cost+Variable\;cost$$

(14) $$Total\;cost\;include\;profit\left(EGP.{hr}^{-1}\right)=Total\;cost\left(EGP.{hr}^{-1}\right)\times Profit\left(\%\right)$$

(15) $$Machine\;production\;cost\;\left(EGP.{ton}^{-1}\right)=\frac{Total\;cost\;include\;profit\;\left(EGP.{hr}^{-1}\right)}{Machine\;productivity\;\left(ton.{hr}^{-1}\right)}$$

## 3 Results and discussion

### 3.1 Forage machine productivity

**Fig. 3:**
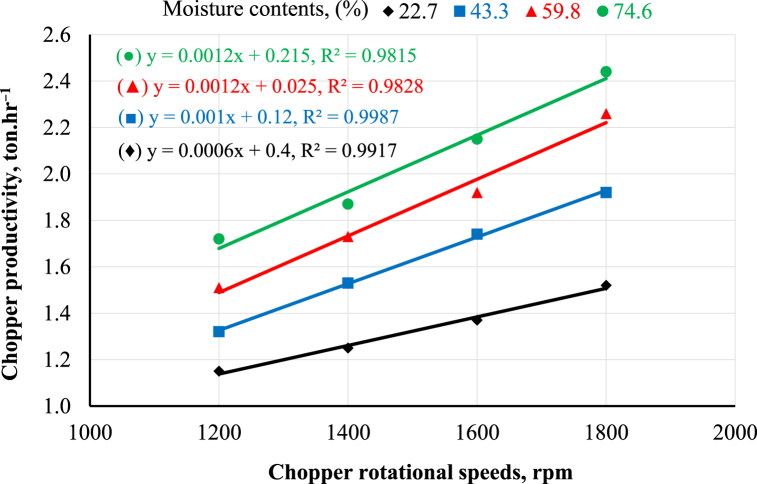
Chopper productivity as affected by chopper rotational speed and moisture content.

**Fig. 4:**
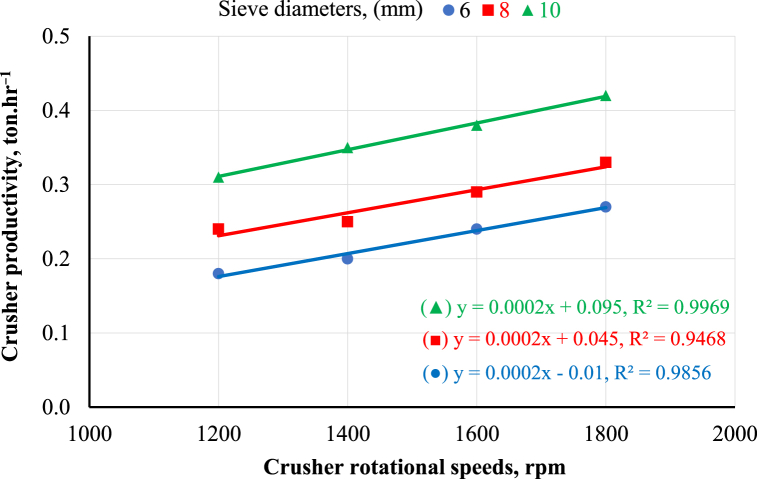
Crusher productivity as affected by crusher rotational speeds and sieve diameters.

Fig. 3, Fig. 4 illustrate the machine's productivity as influenced by the choppers' rotational speeds from 1200 to 1800 rpm and moisture content from 22.7 to 74.8%, and as affected by the crushers' rotational speeds from 1200 to 1800 rpm, and the diameters of sieve holes from 6 to 10 mm. Regarding the effect of the chopper's rotational speed on the machine productivity, as shown in Fig. 3, increasing the chopper's rotational speed increased the machine productivity at any moisture content. It was observed that when the rotational speed of the chopper increased from 1200 to 1800 rpm, the chopper productivity increased from 1.72 to 2.44 ton.hr^−1^ at a moisture content of 74.6%. This may be attributed to increasing chopper rotational speed, which in turn decreased the chopping duration and consequently increased the machine's productivity. These results are in full agreement with Yehia and Omran [22]; Abo-Habaga et al. [23]. The same trend was observed concerning the influence of moisture content on the chopper's productivity, as shown in Fig. 3; increasing moisture content increased chopper productivity at any chopper's rotational speed. The results indicated that increasing moisture content from 22.7 to 74.6% increased the chopper's productivity by 37% at a rotational speed of 1800 rpm. The trend of the obtained results agrees with El Shal and El Didamony [24].

Fig. 4 shows the effect of the crusher's rotational speed on the machine's productivity. The same trend was observed where increasing the crusher's rotational speed increased machine productivity at any sieve diameter. The results revealed that increasing the crusher's rotational speed from 1200 to 1800 rpm increased machine productivity from 0.31 to 0.42 ton.hr^−1^ at a sieve diameter of 10 mm. This may be because increasing the crusher's rotational speed shortened the time required for crushing, which in turn increased the machine's productivity. The previous findings are in agreement with Ahmed [12]. Relating the effect of the sieve diameter on the crusher's productivity, as shown in Fig. 4, increasing the sieve diameter increased the crusher's productivity at any rotational speed. The results indicated that increasing the sieve diameter from 6 to 10 mm increased the crusher's productivity from 0.27 to 0.42 ton.hr^−1^ at a rotational speed of 1800 rpm. This may be because sieves with larger diameters allow crushed maize to discharge more easily than sieves with smaller diameters. These results are in harmony with Ahmed [12]; Ushakov et al. [25]. The statistical analysis of the obtained data was performed using linear regression, as shown in Fig. 3, Fig. 4.

### 3.2 Forage machine efficiency

**Fig. 5:**
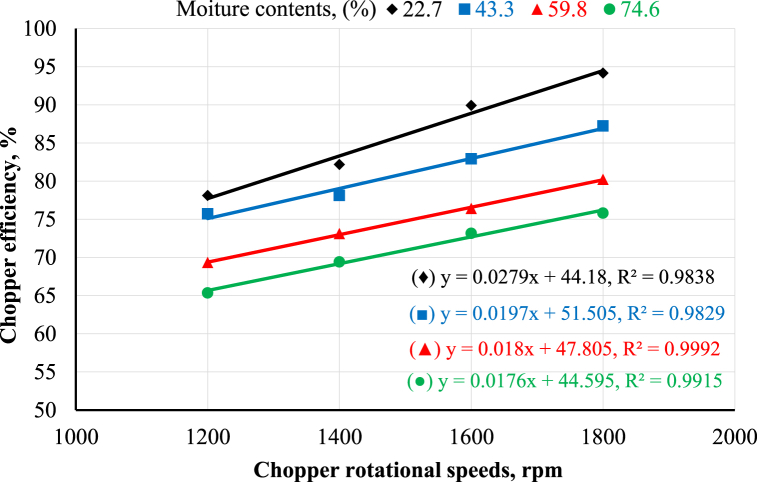
Chopper efficiency as affected by chopper rotational speed and moisture content.

Fig. 5 presents the effect of the chopper's rotational speed on the chopper's efficiency at different moisture contents. It was noted that increasing the chopper's rotational speed increased its efficiency at any moisture content. Fig. 5 shows that increasing the chopper's rotational speed from 1200 to 1800 rpm increased the chopper's efficiency from 78.12 to 94.17, 75.73 to 87.24, 69.34 to 80.23, and 65.36 to 75.82% at moisture contents of 22.7, 43.3, 59.8 and 74.6%, respectively. This may be attributed to the increase in the number of chopper blade beats per unit of time, which causes an increase in the mass of the optimal forage cutting length. The trend of the obtained results agrees with El Shal and El Didamony [24]. In contrast, the influence of moisture content on the chopper's efficiency followed the opposite trend. As shown in Fig. 5, increasing moisture content decreased the chopper's efficiency at any rotational speed. The results indicated that increasing moisture content from 22.7 to 74.6% decreased the chopper's efficiency by percent of 16.3, 15.5, 18.6, and 19.5% at rotational speeds of 1200, 1400, 1600, and 1800 rpm, respectively. This may be attributed to increasing cutting resistance because of increased moisture content. These results are in line with Abo-Habaga et al. [23].

**Fig. 6:**
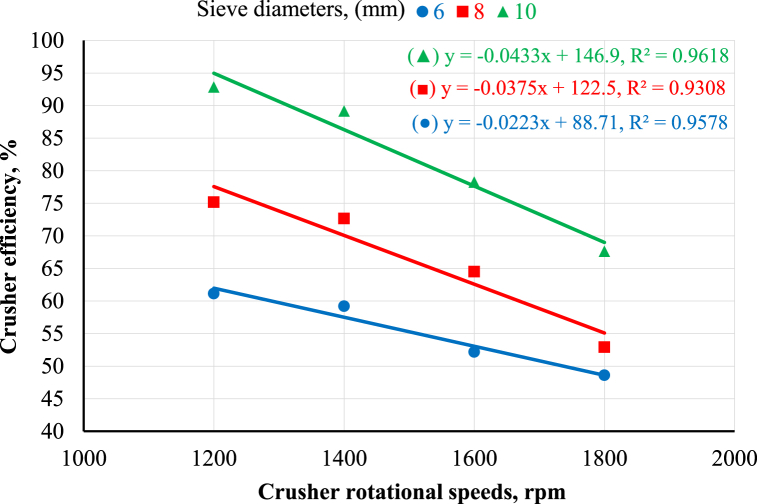
Crusher efficiency as affected by crusher rotational speeds and sieve diameters.

Fig. 6 shows the effect of the crusher's rotational speed on the crusher's efficiency at different sieve diameters. The results clear that increasing the crusher's rotational speed decreased the crusher's efficiency at any sieve diameter. Fig. 6 reveals that increasing the crusher's rotational speed from 1200 to 1800 rpm decreased the crusher's efficiency from 61.13 to 48.62, 75.18 to 52.93, and 92.85 to 67.63% at 6, 8, and 10 mm sieve hole diameters, respectively. This may be because increasing the crusher rotational speed causes the particle diameter of maize ears to decrease below the required diameter, decreasing the crusher efficiency. The obtained results are in agreement with those reported by Ahmed [12]; El Ashry et al. [26]; Okasha [9]; Hegazy et al. [10]. Concerning the effect of sieve diameter on the crusher's efficiency, Fig. 6 reveals that increasing sieve diameters increased crusher efficiency at any rotational speed. The results indicated that increasing the sieve diameter from 6 to 10 mm increased the crusher's efficiency by percent of 34.2, 33.6, 33.3, and 28.1% at rotational speeds of 1200, 1400, 1600, and 1800 rpm, respectively. These results are in agreement with Ahmed [12]. The statistical analysis of the obtained data was performed using linear regression, as shown in Fig. 5, Fig. 6.

### 3.3 Forage machine's specific energy

**Fig. 7:**
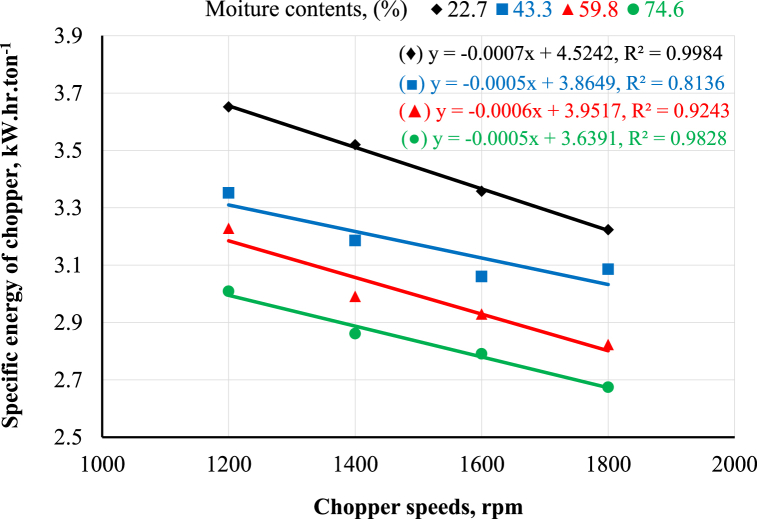
Effect of chopper rotational speed and moisture content on the chopper's specific energy requirements.

Fig. 7 presents the effect of the chopper's rotational speed on the chopper's specific energy under different moisture contents. The general trend demonstrates that increasing the chopper's rotational speed decreased the chopper's specific energy at any different moisture content. The results revealed that increasing the chopper's rotational speed from 1200 to 1800 rpm decreased the chopper's specific energy from 3.65 to 3.22, 3.35 to 3.09, 3.23 to 2.82, and 3.01 to 2.67 kW h.ton^−1^ at moisture contents of 22.7, 43.3, 59.8 and 74.6%, respectively. These results are in harmony with Okasha [9]; El Shal and El Didamony [24]; Awad et al. [11]. Regarding the influence of moisture content on the chopper's specific energy under different rotational speeds, Fig. 7 shows that increasing moisture content decreased the chopper's specific energy speed at any rotational speed. The results demonstrated that increasing moisture content from 22.7 to 74.6% decreased the chopper's specific energy by percent of 17.5, 18.7, 17.0, and 17.1% at rotational speeds of 1200, 1400, 1600, and 1800 rpm, respectively. These results are in line with El Shal and El Didamony [24].

**Fig. 8:**
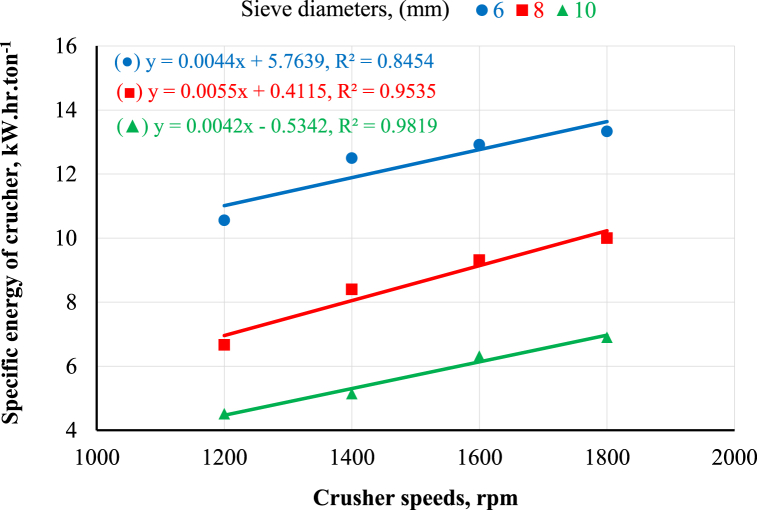
Effect of crusher rotational speed and moisture content on the crusher's specific energy requirements.

Fig. 8 demonstrates the effect of the crusher's rotational speed on the crusher's specific energy under different sieve diameters. It was noted that increasing the crusher's rotational speed increased the crusher's specific energy at any sieve diameter. The results revealed that increasing the crusher's rotational speed from 1200 to 1800 rpm increased the crusher's specific energy from 10.6 to 13.3, 6.7 to 10.0, and 4.5 to 6.9 kW h.ton^−1^ at 6, 8, and 10 mm sieve holes diameter, respectively. Regarding the influence of sieve diameter on the crusher's specific energy under different crusher's rotational speeds, Fig. 8 demonstrates that increasing sieve diameter decreased the crusher's specific energy at any rotational speed. The obtained results revealed that increasing the sieve diameter from 6 to 10 mm decreased the crusher's specific energy by percent of 57.5, 59.2, 51.2, and 48.1% at the crusher's rotational speeds of 1200, 1400, 1600, and 1800 rpm, respectively. This may be attributed to the high productivity and low energy required. These results are in harmony with Ahmed [12]. The statistical analysis of the obtained data was performed using linear regression, as shown in Fig. 7, Fig. 8.

### 3.4 Forage machine's operation cost

**Fig. 9:**
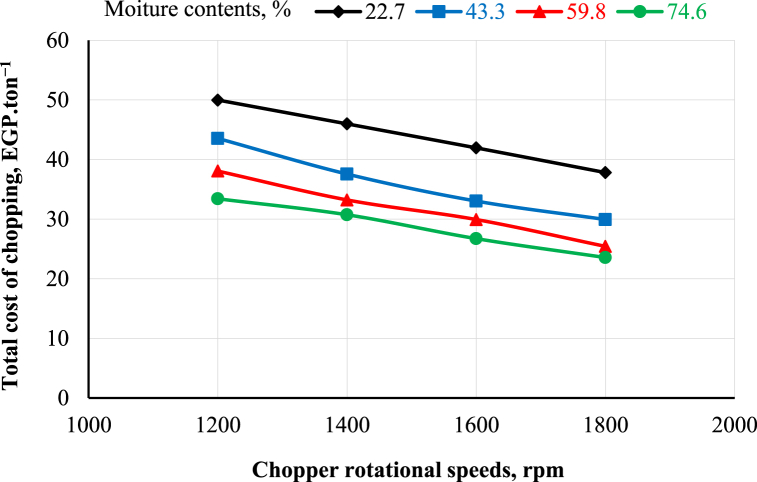
Effect of chopper rotational speed and moisture content on the total cost of chopping.

**Fig. 10:**
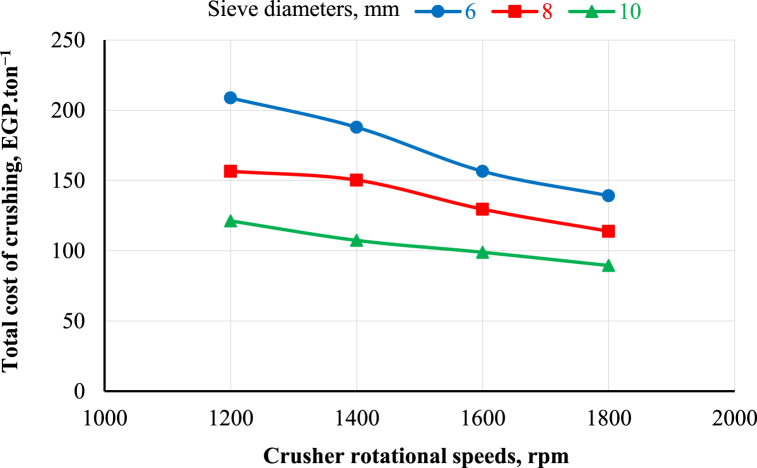
Effect of crusher rotational speed and moisture content on the total cost of crushing.

Fig. 9, Fig. 10 show the effects of rotational speed, moisture content and sieve diameter on the total cost of chopping and crushing operations, respectively. The results reveal that the total cost of chopping decreases with increasing the chopper's rotational speed and moisture content. It was observed that when the chopper's rotational speed increased from 1200 to 1800 rpm, the total cost of chopping decreased from 49.98 to 37.82, 43.55 to 29.94, 38.07 to 25.43 and 33.42 to 23.56 EGP.ton^−1^ at 22.7, 43.3, 59.8, and 74.6% moisture content, respectively. In addition, the total cost of crushing decreased with increasing the crusher's rotational speed and sieve hole diameter. It was observed that when the crusher's rotational speed increased from 1200 to 1800 rpm, the total cost of crushing decreased from 208.78 to 139.19, 156.58 to 113.88, and 121.23 to 89.48 EGP.ton^−1^ at 6, 8, and 10 mm sieve hole diameter, respectively.

The highest values of total cost were 49.98 and 208.78 EGP.ton^−1^ at 1200 rpm chopper rotational speed and 22.7% moisture content for the chopper and 1200 rpm crusher rotational speed and 6 mm sieve hole diameter for the crusher, respectively. In contrast, the lowest total cost values were 23.56 and 89.48 EGP.ton^−1^ at 1800 rpm chopper rotational speed and 74.6% moisture content for the chopper and 1800 rpm crusher rotational speed and 10 mm sieve hole diameter for the crusher, respectively.

## 4 Conclusion

The trials were conducted to develop, manufacture, and evaluate a local chopping and crushing machine made from local raw materials. The developed chopping and crushing machine was able to lower operating costs, and it was easy and simple to handle. The obtained results recommended that the optimum efficiencies were 94.17 and 92.85% for the chopper and crusher were observed at the chopper's rotational speed of 1800 rpm, moisture content of 22.7%, crusher's rotational speed of 1200 rpm, and sieve hole diameter of 10 mm, respectively. The machine productivity of 2.44 and 0.31 ton.hr^−1^, the required specific energy of 3.22 and 4.5 kW h.ton^−1^, and the estimated production costs of 23.56 and 121.24 EGP.ton^−1^ (1.25 and 6.38 USD.ton^−1^) were obtained for the chopper and the crusher, respectively at the previous optimum chopper and crusher efficiencies. The main limitations of the designed chopping and crushing forage machine are that the developed machine can not be used for mass production to serve larger areas in its current figure, skilled labor is required to operate it, so that it may require light training at the beginning of operations, the range of local materials that can be used in manufacturing is limited, with fixed machine position, the additional cost to transfer the maize stalks will be applied. Currently, the dual-purpose developed machine is suitable for small and medium farms, and our future perspective for this machine is pursuing amending it to fit large-scale farms. Moreover, the possibility of using solar energy as an alternative power source will give us the privileges the versatility of using the developed machine with different forage crops in the open field.

## Author contribution statement

Hossam El Ghobashy; Yousry Shaban; El-Sayed Khater: Conceived and designed the experiments; Contributed reagents, materials, analysis tools or data; Wrote the paper.

Mahmoud Okasha; Solaf Abd El-Reheem; Mohamed Abdelgawad: Conceived and designed the experiments; Performed the experiments; Contributed reagents, materials, analysis tools or data; Wrote the paper.

Rania Ibrahim; Heba Ibrahim; Khaled Abdelmohsen: Performed the experiments; Analyzed and interpreted the data; Wrote the paper.

Mahmoud Awad; Mokhtar Cottb; Mohamed Elmeadawy; Wael Fathy: Conceived and designed the experiments; Analyzed and interpreted the data; Contributed reagents, materials, analysis tools or data.

## Data availability statement

Data will be made available on request.

## Funding statement

This research did not receive any specific grant from funding agencies in the public, commercial, or not-for-profit sectors.

## Declaration of competing interest

The authors declare no conflict of interest.
